# Calcaneal quantitative ultrasound and Phalangeal radiographic absorptiometry alone or in combination in a triage approach for assessment of osteoporosis: a study of older women with a high prevalence of falls

**DOI:** 10.1186/1471-2318-14-143

**Published:** 2014-12-20

**Authors:** Katja Thomsen, Jesper Ryg, Anne P Hermann, Lars Matzen, Tahir Masud

**Affiliations:** Institute of Clinical Research, University of Southern Denmark, Odense, Denmark; Department of Geriatric Medicine, Odense University Hospital, Odense, Denmark; Department of Endocrinology, Odense University Hospital, Odense, Denmark; Nottingham University Hospitals NHS Trust, Nottingham, UK

## Abstract

**Background:**

The objective of this study was to investigate if application of United Kingdom National Osteoporosis Society (UK-NOS) triage approach, using calcaneal quantitative ultrasound (QUS), phalangeal radiographic absorptiometry (RA), or both methods in combination, for identification of women with osteoporosis, would reduce the percentage of women who need further assessment with Dual Energy X-ray Absorptiometry (DXA) among older women with a high prevalence of falls.

**Methods:**

We assessed 286 women with DXA of hip and spine (Hologic Discovery) of whom 221 were assessed with calcaneal QUS (Achilles Lunar), 245 were assessed with phalangeal RA (Aleris Metriscan), and 202 were assessed with all three methods. Receiver operator characteristics (ROC) curve for QUS, RA, and both methods in combination predicting osteoporosis defined by central DXA were performed. We identified cutoffs at different sensitivity and specificity values and applied the triage approach recommended by UK-NOS. The percentage of women who would not need further examination with DXA was calculated.

**Results:**

Median age was 80 years (interquartile range [IQR]) [75–85], range 65–98. 66.8% reported at least one fall within the last 12 months. Prevalence of osteoporosis was 44.4%. Area under the ROC-curve (AUC) (95% confidence interval (CI)) was 0.808 (0.748-0.867) for QUS, 0.800 (0.738-0.863) for RA, and 0.848 (0.796-0.900) for RA and QUS in combination. At 90% certainty levels, UK-NOS triage approach would reduce the percentage of women who need further assessment with DXA by 60% for QUS, and 43% for RA. The false negative and false positive rates ranged from 4% to 5% for QUS and RA respectively. For the combined approach using 90% certainty level the proportion of DXAs saved was 22%, the false negative rate was 0% and false positive rate was 0.5%. Using 85% certainty level for the combined approach the proportion of DXAs saved increased to 41%, but false negative and false positive values remained low (0.5%, and 0.5% respectively).

**Conclusions:**

In a two-step, triage approach calcaneal QUS and phalangeal RA perform well, reducing the number of women who would need assessment with central DXA. Combining RA and QUS reduces misclassifications whilst still reducing the need for DXAs.

## Background

Fractures related to osteoporosis are widely recognized as an important health problem because of their significant morbidity, mortality, and costs. The prevalence of osteoporosis in the European Union is estimated at 27.6 million and is 3–4 times higher in women over the age of 50 than in men [1]. Worldwide osteoporosis results in nearly 9 million fractures annually [2].

Most osteoporotic fractures are preceded by a fall. As with osteoporosis the prevalence of falls among older people is high. One third of the population over 65 years of age fall every year [3–5]. The prevalence of falls increases with age and women are more prone to fall than men [6]. Moreover having a previous fall increases the risk of having a fall in the subsequent year [7]. Several studies have confirmed the association between prior falls or fall related predictors, and osteoporotic fractures [8–11]. The risk of fracture is increased for persons with a prior fall, or osteoporosis. Having both risk factors has an additive effect on the relative risk of fracture [12]. The United Kingdom Guidance on Falls, National Institute of Clinical Excellence (NICE), and other national clinical guidelines emphasize the importance of assessing for osteoporosis in people who present with falls [13–16].

Osteoporosis is defined as a systemic skeletal disease characterized by low bone mass and microarchitectorial deterioration of bone tissue, with a consequent increase in bone fragility and susceptibility to fracture [17]. The operational definition of osteoporosis is based on BMD with a value of BMD 2.5 standard deviations (SD) or more below the young female adult mean characterized as osteoporosis [18]. The current standard method for assessment of BMD is DXA of the hip or spine [14]. DXA devices are relatively expensive and usually require patients to be referred to a hospital-based facility, which makes the method less accessible. A population-based study showed that only 30% of the women who reported a history of falls within the last year had a DXA performed. Furthermore the use of DXA was influenced by the distance to the nearest DXA facility, particularly among women over the age of 65 years old compared to younger women [19]. In addition, suffering from a severe medical condition and poor health has been shown to be associated with non-attendance for DXA [20].

Calcaneal QUS and phalangeal RA are alternative imaging techniques for assessing bone. Calcaneal QUS provides a measurement of bone related to BMD and bone architecture. Compared to DXA, QUS has the advantages of being cheaper, portable, and free of ionizing radiation [21]. RA of the phalanges measures BMD of the middle phalanx of the second, third, and fourth fingers. It is a self-contained system that is small enough to be placed on a desktop, portable, easy to manage, and associated with a low radiation dose [22]. In principle, these techniques can be performed in the emergency room for patients who present with a fall, in the falls clinic as part of the falls risk assessment, or by the general practitioner.

Because the age related decline in mean T-scores at different BMD sites is different for the different techniques used, the World Health Organisation (WHO) T-score definition of osteoporosis should not be used to interpret measures of peripheral bone density measurements [23, 24]. UK-NOS proposes a triage approach for application of peripheral X-ray absorptiometry in the management of osteoporosis. Using device specific thresholds at a level of sensitivity and specificity of 90% for the identification of patients with osteoporosis, patients are categorized as normal, abnormal, or equivocal. Patients with equivocal findings should be referred for further assessment with central DXA for definitive diagnosis [25].

Studies of application of the UK-NOS triage approach to calcaneal QUS and phalangeal RA have suggested a reduction in referral rate for central DXA [23, 26–29]. However, it is not known whether these results are applicable to older people with a high risk of falls. Moreover, the UK-NOS triage approach is associated with a percentage of people falsely classified as osteoporotic or non-osteoporotic. It is unclear if combining different peripheral techniques such as calcaneal QUS and phalangeal RA could reduce this percentage.

The objectives of this study were, in a sample of older women with a high prevalence of falls; firstly, to assess the accuracy of phalangeal RA and calcaneal QUS to detect osteoporosis, defined by low BMD assessed with central DXA; secondly, to examine if application of a triage approach with calcaneal QUS or phalangeal RA reduces the referral rate for central DXA, and finally to examine if application of phalangeal RA and calcaneal QUS in combination reduces the referral rate for central DXA at a lower misclassification rate than using the individual methods alone.

## Methods

This cross sectional cohort study was conducted at the Geriatric Department of Medicine, Odense University Hospital, Denmark. Participants were recruited for the study from May 2012 until November 2013.

### Participants

The study sample was derived from a case control study designed to assess the prevalence of osteoporosis among women who had fallen compared to women with no falls. A total of 322 women participated in the study. We consecutively recruited 117 women from the falls clinic at Odense University Hospital and 205 women from the community. In the community group 114 were aged matched controls with a history of falls in the previous year and 91 were aged matched controls with no history of falls. The women recruited from the community, were randomly selected among women living in the municipality of Odense. Criteria for inclusion in this study were age equal to or above 65 years and female sex. Criteria for exclusion were; not willing to or unable to give informed consent, or not able to be mobilized on to the DXA scan.

Participants were interviewed about risk factors for osteoporosis and falls, co-morbidity, prior fracture, and current medication. Information on co-morbidity and prior fracture was validated from medical records. After the interview, the participants were referred for bone assessments.

### Bone assessments

All bone measurements were performed the same day on the same scanners, by trained personal.

#### Dual energy x-ray absorptiometry

We measured BMD of total hip, femoral neck, and lumbar spine by DXA using Hologic Discovery A device (Hologic Inc.). The T-scores of the hip were calculated using the National Health and Nutrition Examination Survey (NHANES) reference database. The T-scores for the spine were calculated using the manufacturer reference database. Employing the WHO definition of osteoporosis, participants were categorized as osteoporotic when DXA BMD of the femoral neck, total hip, or lumbar spine were lower than 2.5 SD below the young normal mean.

#### Quantitative ultrasound of the calcaneus

QUS of the calcaneus was performed using GE Medical systems Lunar Achilles Insight. The device provides measures of the velocity and frequency attenuation of the sound wave propagation through bone. The measures are termed “speed of sound” (SOS) and “broad band ultrasound attenuation” (BUA). The device also provides a combination of SOS and BUA, called stiffness index (SI) with the associated T-score of the calcaneus. The T-score was derived from the manufacturer reference database, and provided by the device.

#### Phalangeal radiographic absorptiometry

Phalangeal BMD was measured by RA of the middle phalanges of the second, third, and fourth fingers using a compact RA system Aleris Metriscan® (Alera Inc. Fremont). The device provides a measure of BMD expressed in arbitrary units (mineral mass/area), g/m^2^ and a T-score based on the manufacturers reference database.

### Statistical methods

Results are presented as mean and SD, median and i IQR, or percentage, as appropriate. For comparison, we used students t-test, Mann–Whitney- and Chi^2^ test as appropriate. P-values below 0.05 were considered statistical significant. Pearson correlation coefficients (r) were used to evaluate the correlations between the measurements of DXA, QUS, and RA variables. The accuracy of QUS and RA, in terms of the ability to discriminate between osteoporotic and non-osteoporotic women, was evaluated using ROC curves, and calculating AUC. A ROC-curve and AUC for the combination of both tests was derived from a model using logistic regression. We used DXA determined osteoporosis (BMD ≤ -2.5) as the dependent variable, and RA T-score and QUS BUA as independent continual variables. Hosmer and Lemeshow’s goodness-of-fit test was calculated. For the peripheral techniques we derived the optimal cutoff for the diagnosis of osteoporosis by determining the Youden index [30]. We also set cutoffs to identify osteoporosis with sensitivities or specificities of 90% and 95% and calculated the corresponding sensitivity, specificity, negative and positive predictive values (NPV, PPV). We applied a triage approach as recommended by UK-NOS with a certainty level of 90%, and a more restricted approach with a certainty level of 95%. The number of people, who would not need further assessment with DXA according to these triage approaches, was calculated for both. A dot diagram to show the distribution of the measures of calcaneal QUS and phalangeal RA between osteoporotic and non-osteoporotic participants was created, with horizontal lines corresponding to the upper and lower thresholds at 90% sensitivity and specificity, respectively. Finally, we evaluated a model for combining the two peripheral bone assessment techniques. At the cutoffs corresponding to a predetermined sensitivity of 90% for each method, we categorized each person as osteoporotic or not osteoporotic. We applied the two tests in parallel using the “OR-rule”, a positive result in either test, RA or QUS, would classify the person as osteoporotic. The same procedure was followed for cutoffs at a predetermined specificity of 90%. We applied the “AND-rule”, both tests had to be positive in order to classify the person as osteoporotic [31] . We then applied the UK-NOS triage approach to the combined test. Those classified as non-osteoporotic in both tests at the cutoffs corresponding to 90% sensitivity were classified as normal. Those classified as osteoporotic in both tests at the cutoffs corresponding to 90% specificity were classified as abnormal. Those who were not classified according to these two approaches were classified as equivocal. The same procedure was followed at cutoffs corresponding to 85% and 95% certainty levels.

Ethical permission for this study was granted by the Regional Ethics Committee of Southern Denmark (S-20120262) and written informed consent was given by the participants. The Danish Data Protection Agency approved the study (2008-58-0035) and the study was registered at ClinicalTrial.gov (NCT01600547).

## Results

A total of 286 women were assessed with DXA of hip, spine, or both, and measurement with calcaneal QUS, phalangeal RA, or both. Two hundred forty-five (86%) women were assessed with phalangeal RA, 221 (77%) were assessed with QUS. A total of 202 (71%) women were assessed with DXA and both peripheral scans. DXA results for the spine were missing for 11 women: The vertebrae were not suitable for diagnosis because of severe spondylarthrosis. DXA results for the hip were missing for 11 women because of bilateral hip-prosthesis. Phalangeal RA was missing in five women because the hand could not be placed flat on the platform due to severe arthritis, and in one woman because she wore rings that were not removable. 37 women were not assessed with phalangeal RA and 66 women were not assessed with calcaneal QUS due to technical problems. The participants not having either RA or QUS (n = 84) did not differ in age or prevalence of osteoporosis compared to the total study sample.

Median [IQR] age of the women participating in the study was 80 years [75–85], range 65–98 years. The prevalence of osteoporosis by DXA at any site (spine, femoral neck, or total hip) was 44.4%. 66.8% reported at least one fall within the last 12 months. The characteristics of the osteoporotic and non-osteoporotic women are shown in Table 1. The proportion of women with a prior fracture after the age of 50 was significantly higher among women with osteoporosis compared to women without osteoporosis (53.5% vs. 38.4%). Body mass index (BMI) was lower (25.2 kg/m^2^ vs. 27.1 kg/m^2^) and age was higher (82 years vs. 78 years) among osteoporotic women. The groups did not differ regarding the number of medications, diagnoses, and the proportion of women with one or more falls within the last 12 months. The measures of calcaneal QUS and phalangeal RA were significantly lower among DXA-defined osteoporotic women (Table 1).

**Table 1:** **Characteristics of the study sample**

	Without osteoporosis (n = 159)	With osteoporosis (n = 127)	p value
Age, years median [IQR] (range)	78 [74–89] (65–93)	82 [76–88] (65–98)	<0.001^1^
BMI, kg/m^2^ median [IQR]	27 [24–32]	25 [22–29]	<0.001^1^
No. of medications median [IQR]	5 [3–7]	5 [3–7]	0.76^1^
No. of diagnoses median [IQR]	3 [2–4]	3 [2–5]	<0.05^1^
Prior fracture after the age of 50%	39	54	<0.01^3^
≥one falls within the last 12 months%	69.8	63.3	0.24^3^
DXA T-score (n = 275)			
Lumbar spine median [IQR]	-1.07 [-1.66–0.0]	-2.68 [-3.16– –2.04]	<0.001^1^
Femoral neck median [IQR]	-1.68 [-2.06– –1.0]	-2.90 [-3.23– –2.51]	<0.001^1^
Total hip median [IQR]	-1.07 [-1.60– –0.42]	-2.35 [-2.9– –1.89]	<0.001^1^
QUS calcaneal (n = 221)			
BUA dB/MHz, median [IQR]	105.6 [95.2–117.8]	86.9 [78.9–96.7]	<0.001^1^
SOS m/s, median [IQR]	1539.3 [1519.4–1558.3]	1508.3 [1488.1–1527.6]	<0.001^1^
SI T-score, median [IQR]	-1.2 [-2.1– –0.3]	-2.8 [-3.6– –2.0]	<0.001^1^
RA phalangeal (n = 245)			
T-score median [IQR]	-1.36 [-2.08– –0.37]	-2.62 [-3.36– –1.90]	<0.001^1^

*Abbreviations: IQR* Inter Quartile Range, *BMI* Body Mass Index, *DXA* Dual Energy X-ray Absorptiometry, *QUS* Quantitative Ultrasound, *RA* Radiographic Absorptiometry, *BUA* Broadband Ultrasound Attenuation, *SOS* Speed of Sound, *SI* Stiffness Index.^1^Man-Whitney test, ^2^two sample t-test, ^3^chi^2^ test.

The correlation coefficients between peripheral measures and central DXA ranged from 0.36 to 0.63. The highest correlation was between BUA and BMD of the total hip (Table 2). Adjusting for height and weight did not meaningfully affect correlation coefficients.

**Figure 1:**
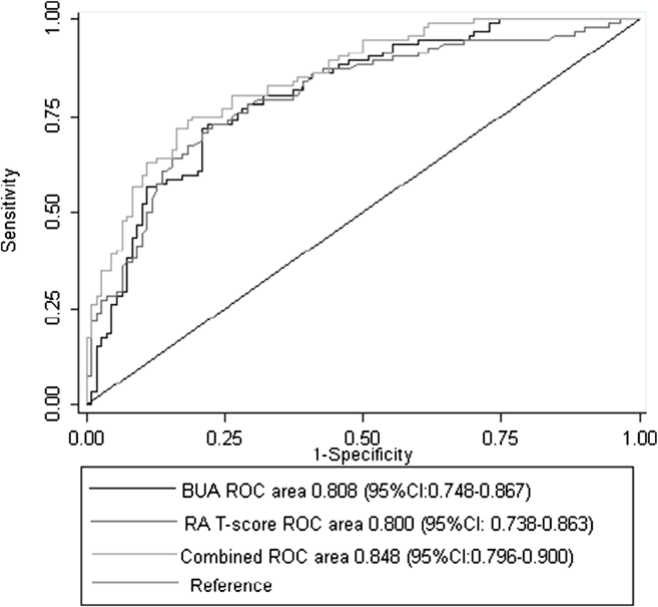
**Receiver operator characteristics (ROC) curves.** ROC-curves for calcaneal QUS (BUA), phalangeal RA (RA T-score), and a combination of BUA and RA T-score (combined) for discrimination between osteoporotic and non-osteoporotic individuals. Hosmer and Lemeshow’s goodness-of-fit test: P = 0.61. Abbreviations: QUS = Quantitative Ultrasound, RA = Radiographic Absorptiometry, BUA = Broadband Ultrasound Attenuation.

Among the different calcaneal QUS variables, BUA performed best in the ROC-curve analysis and our further analysis are therefore restricted to BUA. The ROC-curves for BUA, RA T-score, and the combined test (BUA and RA T-score) are shown in Figure 1. The AUCs of the measurements of calcaneal QUS and the phalangeal RA ranged from 0.800 to 0.848 (Figure 1).

**Figure 2:**
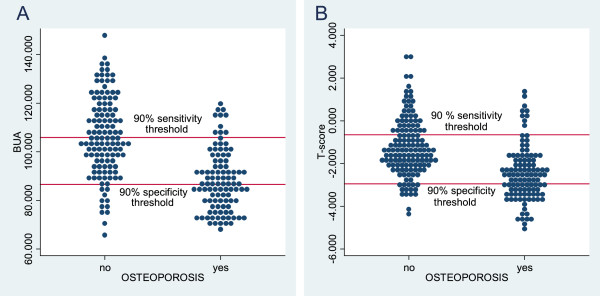
**The distribution of the results of calcaneal QUS (A) and phalangeal RA (B).** Plots showing the distribution of the results of QUS of the calcaneus **(A)** and phalangeal RA **(B)**. Horizontal lines represent the upper and lower triage thresholds at a 90% certainty level. **A**: 90% sensitivity threshold: BUA=105.88, 90% specificity threshold: BUA=86.63. **B**: 90% sensitivity threshold: T-score= -0.65, 90% specificity threshold T-score=-2.95. Abbreviations: QUS = Quantitative Ultrasound, RA = Radiographic Absorptiometry, BUA = Broadband Ultrasound Attenuation.

The distribution of the values of BUA and RA T-score within osteoporotic and non-osteoporotic participants is shown in Figure 2. The horizontal lines reflect the upper and lower thresholds corresponding to the 90% certainty level.

**Table 2:** **Correlations between calcaneal QUS measures, phalangeal RA T-score, and central DXA**

Pearsons correlation	QUS BUA	QUS SOS	QUS SI T-score	RA T-score	DXA femoral neck BMD	DXA total hip BMD	DXA lumbar spine BMD
QUS BUA	1.00						
QUS SOS	0.73	1.00					
QUS SI T-score	0.94	0.91	1.00				
RA T-score	0.49	0.43	0.50	1.00			
DXA femoral neck BMD	0.48	0.36	0.46	0.42	1.00		
DXA total hip BMD	0.63	0.51	0.62	0.43	0.83	1.00	
DXA lumbar spine BMD	0.43	0.39	0.45	0.38	0.42	0.59	1.00

Pearson’s correlations coefficient between calcaneal QUS measures, phalangeal RA and central DXA. *Abbreviations: DXA* Dual Energy X-ray Absorptiometry, *QUS* Quantitative Ultrasound, *RA* Radiographic Absorptiometry, *BUA* Broadband Ultrasound Attenuation, *SOS* Speed of Sound, *SI* Stiffness Index.

### Calcaneal QUS (Table 3)

**Table 3:** **Accuracy of calcaneal QUS in predicting osteoporosis**

Calcaneal QUS (n = 221)	Youden ^1^	UK-NOS triage approach 90% certainty level	UK-NOS triage approach 95% certainty level
BUA upper/lower cutoff	93.88	105.88/86.63	114.5/80.09
Sensitivity (95% CI)	74.0 (64.3–82.3)	90.0 (82.7–95.1)	95.0 (88.7–98.4)
Specificity (95% CI)	78.5 (70.1–85.5)	90.1 (83.3–94.8)	95.0 (89.5–98.2)
PPV (95% CI)	74.0 (64.3–82.3)	80.6 (68.6–89.6)	83.3 (67.2–93.6)
NPV (95% CI)	78.5 (70.1–85.5)	85.7 (74.3–92.9)	88.1 (74.4–96)
False negative n (%)	26 (11.8)	10 (4.5)	5 (2.3)
False positive n (%)	26 (11.8)	12 (5.4)	6 (2.7)
DXA scans avoided n (%)	NA	132 (59.7)	78 (35.3)

Accuracy of calcaneal QUS in predicting osteoporosis applying the optimal cutoff and UK-NOS triage approach at 90% and 95% certainty levels. ^1^The optimal cutoff calculated according the Youden index [30]. *Abbreviations: QUS* Quantitative Ultrasound, *BUA* Broadband Ultrasound Attenuation, *UK-NOS* United Kingdom National Osteoporosis Society, *PPV* Positive Predictive Value, *NPV* Negative Predictive Value, *DXA* Dual Energy X-ray Absorptiometry, *CI* Confidence Interval, *NA* not available.

The optimal cutoff, corresponding to the Youden index was 93.88 dB/MHz for BUA. At this cutoff sensitivity and specificity was 74.0% and 78.5%, respectively. PPV and NPV values were 74.0% and 78.5%, respectively. At the upper cutoffs defined at sensitivities of 90% and 95% (BUA = 105.88 dB/MHz, BUA = 114.50 dB/MHz), the corresponding specificities were 49.6% and 30.6% (not shown in table). The percentages of women falsely classified as non-osteoporotic were 4.5% and 2.3%. Defining lower cutoffs at specificities of 90% and 95% respectively (BUA = 86.63 dB/MHz, BUA = 80.09 dB/MHz), the corresponding sensitivities were 50.0% and 30.0% (not shown in table). The percentages of women falsely diagnosed as osteoporotic were 5.4% and 2.7%. Applying the triage approach at certainty levels of 90% and 95% respectively, 59.7% or 35.3% of the study sample would not need further assessment with DXA and 9.9% (4.5% + 5.4%) or 5.0% (2.3% + 2.7%) of the women would be misclassified as either osteoporotic or non-osteoporotic. Comparing subgroups of women presenting with at least one fall (n = 135) with women who did not have a fall (n = 86) the percentage of DXA’s saved, at a 90% certainty-level was 60% and 60.5% respectively, misclassification-rates were 10.4% and 10.5%, respectively. At a 95% certainty-level the corresponding numbers were 36.3% and 33.7% respectively, misclassification-rates were 6.7% and 2.3%, respectively.

### Phalangeal RA (Table 4)

**Table 4:** **Accuracy of phalangeal RA in predicting osteoporosis**

Phalangeal RA (n = 245)	Youden ^1^	UK-NOS triage approach 90% certainty level	UK-NOS triage approach 95% certainty level
RA T-score Upper/lower cutoff	-2.22	-0.65/–2.95	0.28/–3.32
Sensitivity (95% CI)	67.6 (57.9–76.3)	90.7 (83.6–95.5)	95.4 (89.5–98.5)
Specificity (95% CI)	78.1 (70.2–84.7)	90.5 (84.3–94.9)	95.6 (90.7–98.4)
PPV (95% CI)	70.9 (61.1–79.4)	75.0 (61.1–86)	82.9 (66.4–93.4)
NPV (95% CI)	75.4 (67.4–82.2)	81.1 (68–90.6)	77.3 (54.6–92.2)
False negative n (%)	35 (14.3)	10 (4.1)	5 (2.0)
False positive n (%)	30 (12.2)	13 (5.3)	6 (2.5)
DXA scans avoided n (%)	NA	105 (42.9)	57 (23.3)

Accuracy of phalangeal RA in predicting osteoporosis applying the optimal cutoff and UK-NOS triage approach at 90% and 95% certainty levels. ^1^the optimal cutoff calculated according the Youden index [30]. *Abbreviations: RA* Radiographic Absorptiometry, *UK-NOS* United Kingdom National Osteoporosis Society, *PPV* Positive Predictive Value, *NPV* Negative Predictive Value, *DXA* Dual Energy X-ray Absorptiometry, *CI* Confidence Interval, *NA* not available.

For phalangeal RA the optimal cutoff, corresponding to the Youden index was a T-score of -2.22. At this cutoff, the sensitivity was 67.6% and the specificity was 78.1%. PPV and NPV was 70.9% and 75.4%, respectively. Defining upper-level cutoffs at sensitivities of 90% or 95% (T-score = -0.65 or T-score = 0.28), the corresponding specificities were 31.4% and 12.4% (not shown in table). The proportion of women falsely diagnosed as not osteoporotic was 4.1% and 2.0%. At lower cutoffs defined from specificity of 90% and 95% (T-score = -2.95 or T-score = -3.32), the sensitivity was 36.1% and 26.9% (not shown in table), respectively. The percentage of women falsely classified as not osteoporotic was 5.3% and 2.5%. Applying the triage approach at certainty levels of 90% and 95% respectively, 42.9% or 23.3% of the women would not need further assessment with central DXA, and 9.4% (4.1% + 5.3%) or 4.5% (2.0% + 2.5%) being misclassified as either osteoporotic or non-osteoporotic (Table 4). Comparing subgroups of women presenting with at least one fall (n = 158) with women who did not have a fall (n = 87) the percentage of DXA’s saved, at a 90% certainty-level was 41.8% and 46.0% respectively, misclassification-rates were 11.4% and 5.7%, respectively. At a 95% certainty-level the corresponding numbers were 21.5% and 26.4% respectively, misclassification-rates were 5.1% and 3.4%, respectively.

### The combined test (Table 5)

**Table 5:** **Application of UK-NOS triage approach combining phalangeal RA and calcaneal QUS**

Combined test phalangeal RA and Calcaneal QUS (n = 202)	UK-NOS triage approach 85% certainty level	95% CI	UK-NOS triage approach 90% certainty level	95% CI	UK-NOS triage approach 95% certainty level	95% CI
RA T-score, upper/lower cutoff	-1.36/–2.52	NA	-0.65/–2.75	NA	0.28/–3.32	NA
QUS BUA, upper/lower cutoff	101.74/89.63	NA	105.88/86.63	NA	114.5/80.09	
Sensitivity (%)	98.9	94.1–100	100	96.1–100	100	96.1–100
Specificity (%)	95.5	89.7–98.5	99.1	95.0–100	100	96.7–100
PPV (%)	87.2	72.6–95.7	94.7	74.0–99.9	100	54.1–100
NPV (%)	97.5	86.8–99.9	100	86.3–100	100	75.3–100
False negative (%)	0.5	0.01–2.7	0	0–0.2	0	0–3.2
False positive (%)	2.5	0.8–5.7	0.5	0.01–2.7	0	0–3.9
DXA scans avoided (%)	41.1	34.2-48.2	21.8	16.3–28.1	9.4	6.2–14.9

Application of UK-NOS triage approach combining phalangeal RA and calcaneal QUS at a certainty level of 85%, 90%, or 95% for each method. *Abbreviations:* *QUS* Quantitative Ultrasound, *BUA* Broadband Ultrasound Attenuation, *RA* Radiographic Absorptiometry, *UK-NOS* United Kingdom National Osteoporosis Society, *PPV* Positive Predictive Value, *NPV* Negative Predictive Value, *DXA* Dual Energy X-ray Absorptiometry, *CI* Confidence Interval, *NA* not available.

Combining calcaneal QUS and phalangeal RA, with the cutoffs of each device set at 90% sensitivity, would lead to a NPV of 100%. Applying the cutoffs corresponding to 90% specificity for each method, PPV is 94.7%. Applying the triage approach to the combined test, at the sensitivities and specificities of 90%, the proportion of the study sample who would not need assessment with central DXA was 21.8%. The proportion of false negative and false positive outcomes would be 0% and 0.5%, respectively. At a 95% certainty level for each device, the proportion who would not need central DXA was 9.4%, PPV and NPV was 100%, and misclassification rate was 0% (Table 5).

Because the proportion of DXAs saved was much lower with the combined approach compared to the peripheral techniques (for 90% certainty; 21.8% compared to 59.7% and 42.9%), and the misclassification rate was only 0.5%, we explored the combined approach using the 85% certainty level. For a combined approach at an 85% certainty level for each device, the proportion who would not need central DXA was 41.1%, misclassification rate was 3.0% (Table 5).

## Discussion

This study shows that in a sample of older women, the correlation between DXA BMD of the hip, spine, QUS of the calcaneus, or RA of the phalanges is moderate and the accuracy of calcaneal QUS or phalangeal RA to predict osteoporosis defined by DXA of the hip or spine is moderate. However, the methods perform well applying the triage approach at a 90% certainty level, as recommended by UK-NOS, to each method separately, and when combining the two methods. Referral rate for central DXA is reduced by 60% by calcaneus QUS and by 43% with phalangeal RA. Combining both methods at a certainty level of 90%, would lead to the savings of 22% DXA-scans. In the combined approach the percentage of DXAs saved were increased to 41% by decreasing the certainty level to 85% whilst keeping the misclassification rate acceptably low (false negative 0.5% and false positive 2.5%).

Using the triage approach the selection of cutoff is a trade-off between the number of DXAs avoided and the number of patients being misclassified. Misclassification leads to either over- or undertreatment of osteoporosis. Overtreatment results in unnecessary costs related to the treatment but it also has several consequences related to potential adverse effects to the medical treatment. Atypical femoral fracture or osteonecrosis of the jaw are examples of rare but severe adverse effects due to treatment with bisphosphonate. Under-treatment on the other hand, might result in otherwise preventable fractures with the severe implications this might have to patients and the society.

The drawbacks of the UK-NOS triage approach are that up to 20% are misclassified at the suggested 90% certainty level. Our study shows that increasing the certainty level to 95% decreases the misclassification rate to 5%, at the expenses of less reduction in the referral rate for DXA. Combining QUS and RA in a triage approach at 90% certainty level the misclassification rate is almost zero but at the expense of reducing the proportion of DXAs saved. By reducing the certainty level to 85% in the NOS triage approach the proportion of DXAs saved remained high whilst preserving an acceptably low misclassification rate.

The accuracy of calcaneal QUS has been widely studied. However, the studies vary in terms of the method and the measurement of QUS used. Several studies using Achilles Lunar have shown similar correlation and AUC as our results [32–35]. Application of the UK-NOS triage approach has been studied in different settings. The EPIDOS-study; a population based study of 5,954 elderly women (+75 years), showed that applying the UK-NOS triage approach resulted in the avoidance of 44% DXAs, while 11% were categorised as false positive and 13% false negative [23, 26]. Clowes *et al.* studied the usefulness of the triage approach for several different peripheral devices [27]. Five hundred postmenopausal women recruited from general practitioner aged between 55 and 88 years and 279 women, same age but who recently had a fracture were studied. They found that at a certainty level of either 90% or 95%, between 30% and 60% would not need further examination with DXA. However, osteoporosis was defined only according to DXA of the hip, and the study did not report the percentages of subjects misclassified. Harrison *et al.* showed that in a sample of women aged 50 to 70 years who were referred for a routine bone densitometry scan, at a certainty level of 90%, nearly 50% would not need further assessment with DXA [28]. A cost effective analysis was performed and they concluded that despite the number of DXA’s saved, the cost of unnecessary treatment exceeded the savings attributed to lower fraction of DXA scans. However, the costs of treatment did not correspond to the costs of today and they did not consider the cost of fractures to those misclassified as not osteoporotic.

Prior accuracy studies of the phalangeal RA using the Aleris Metriscan®, have shown correlation coefficients ranging from 0.46-0.66, and AUC ranging from 0.75-0.85. The method has been studied in different settings, men only, people presenting with a prior low energy fracture, women with intermediate or high 10-year fracture risk according to WHO Fracture Risk Assessment Tool (FRAX®), and patients undergoing routine DXA [22, 29, 36–39]. The UK-NOS triage approach, using the 90% certainty level has also been applied to the method. Thorpe *et al.* demonstrated that the referral rate for DXA could be reduced to 44-48%, with 10% being misclassified [22]. Dhainaut *et al.* demonstrated a referral rate of 34%, but did not report the number of individuals misclassified and Friss-Holmberg demonstrated a referral rate of 45%, with 9% being misclassified [29, 39].

The UK-NOS triage approach with phalangeal RA and calcaneal QUS in combination has not been previously studied. Phalangeal RA and calcaneal QUS do not identify exactly the same people with osteoporosis. Combining the methods increases the certainty of the individual correctly classified as either osteoporotic or not osteoporotic. Clinicians would be reassured by using the combined approach because of the negligible false positive and false negative rates for diagnosing osteoporosis. Although an advantageous low misclassification rate, at the 90% certainty level planners of osteoporosis services may find the numbers of scans saved too low. However, using the 85% certainty level the numbers of scans saved remains acceptable but the cost effectiveness of these approaches needs further evaluation.

This study has some limitations. Firstly, we only included women older than 65 years old and therefore the results may not be applicable to younger women and men. Secondly, we included a proportion of women who were non-fallers. Nevertheless, our data do not show any significant differences in the results between fallers and non-fallers. Subgroup analyses did not reveal meaningful differences in DXAs saved and misclassification rates between fallers and non-fallers.

Our study also had several strengths. Firstly, this is the first study to test the accuracy and ability to reduce the referral rate to DXA of calcaneal QUS and phalangeal RA in a sample of older women with a high prevalence of falls. The results are therefore implementable to a clinical setting of diagnosing osteoporosis among older women presenting with falls. Secondly, sample size is large enough to ensure that the true sensitivity and specificity with 95% confidence do not fall below 80%. Thirdly, we suggest a method for combining the two peripheral bone scans and thereby almost eliminating the number of false negatives and false positives. Ideally the thresholds proposed in this paper should be verified in another cohort of older people presenting with falls.

## Conclusion

In a two-step, triage approach calcaneal QUS and phalangeal RA perform well, considerably reducing the number of women who would need assessment with central DXA. Combining RA and QUS in a triage approach reduced misclassifications.
